# The impact of family residence structure on adolescents’ non-cognitive abilities: evidence from China

**DOI:** 10.3389/fpsyg.2024.1367308

**Published:** 2024-04-23

**Authors:** Bin Tang, Siyi Xiao, Yi Zhang, Siyan Liu, Xiaoli Lin, Han Liu

**Affiliations:** ^1^Faculty of Education, Center for Experimental Economics in Education (CEEE), Shaanxi Normal University, Xi’an, China; ^2^School of Public Economics & Administration, Shanghai University of Finance & Economics, Shanghai, China

**Keywords:** family residence structure, skip-generation co-residence, three-generation coresidence, non-cognitive abilities, adolescents

## Abstract

**Purpose:**

The family residence structure serves as a crucial pathway through which the family environment influences adolescents’ development.

**Methods:**

Drawing on nationally representative data, this study employs multiple linear regression models and propensity score matching to examine the impact of various family residence structures on adolescents’ non-cognitive abilities. Causal identification is achieved through propensity score matching, while robustness is assessed using methods such as augmented inverse probability weighting and placebo tests. Heterogeneity analysis is conducted based on gender and household registration, aiming to explore the mechanisms by which family residence structure affects adolescents’ non-cognitive abilities.

**Results:**

The findings indicate that compared to two-parent co-residence households, three-generation co-residence families have significantly positive effects on emotional stability, conscientiousness, and agreeableness among adolescents. In contrast, skip-generation coresidence families exhibit significant negative effects on emotional stability and agreeableness in adolescents. Further investigation into the underlying mechanisms reveals that parental involvement and family socioeconomic status within three-generation co-residence families positively influence adolescents’ non-cognitive abilities.

**Conclusion:**

This study highlights the importance of considering grandparents’ role in adolescent growth and advocates for policy recommendations focusing on enhancing non-cognitive abilities in adolescents from skip-generation co-residence families.

## 1 Introduction

The family serves as a crucial context for the socialization of adolescents (Carman and Zhang, 2012). The structure of residential patterns within the family determines the composition of household members, frequency of communication among family members, and primary guardian for the child. As urbanization accelerates and market economy develops, individuals’ working intensity has been steadily increasing (Perry-Jenkins and Gerstel, 2020). Concurrently, child rearing is becoming more sophisticated and costly (Honda et al., 2019; Robb, 2019). In China, where the social security system is not yet fully developed and home-based services are not extensively commercialized, involving grandparents in child rearing has emerged as a primary strategy to address home-based child rearing issues (Wu and Feng, 2020).

The involvement of grandparents in the care of grandchildren inevitably affects the structure of household residence. While numerous studies have examined the influence of grandparent caregiving on adolescents’ development, the focus has predominantly been on aspects such as adolescent health and academic achievement (Pong and Chen, 2010; Zeng and Xie, 2014; He et al., 2018), with insufficient attention given to non-cognitive abilities. Pong and Chen (2010) found a significant positive effect of co-residence with grandparents on adolescents’ academic performance using samples from Taiwan. Analyzing data from the 2002 China Rural Household Income Survey, Zeng and Xie (2014) discovered that the impact of grandparent-grandchild co-residence on school dropout was only significant when grandparents and grandchildren lived together. Furthermore, utilizing data from the China Health and Nutrition Survey (CHNS), He et al. (2018) determined that grandparent-grandchild co-residence substantially increased the likelihood of childhood obesity. Existing research on the influence of grandparent-grandchild co-residence on adolescents’ non-cognitive skills has not reached a consensus conclusion (Xing and Zhang, 2020; Tian and Zhou, 2021).

The involvement of grandparents in caregiving for grandchildren is a prevalent phenomenon within Chinese families as well as families from developing nations (Falbo, 1991; Wu and Feng, 2020; O’Callaghan et al., 2023). However, existing research on intergenerational living arrangements has paid limited attention to comparing three-generation co-residence (where grandparents, parents, and grandchildren live together) with skip-generation co-residence (where only grandparents and grandchildren live together). These distinct family residence structures exhibit systematic differences: the former emphasizes the supplementary role of grandparents to parents, while the latter leans toward substituting parental responsibilities (Wikle and Hoagland, 2020; Yang and Wild, 2022). In order to provide a clearer illustration of the three family living structures, namely two-parent co-residence, three-generation co-residence, and skip-generation co-residence, Table 1 is utilized to demonstrate the differences in residential structure among these household types. Therefore, this study aims to compare the effects of three-generation co-residence and skip-generation co-residence on non-cognitive abilities among adolescents within the same sample. This comparison is crucial for comprehending how different family residence structures involving living with grandparents impact adolescent development.

**Table 1:** Types of family residence structure.

Types of residence	Family members who live together
Two-parent co-residence	Adolescent(s); parents
Skip-generation co-residence	Adolescent(s); grandparents
Three-generation co-residence	Adolescent(s); parents; grandparents

Numerous studies have extensively investigated the substantial impact of non-cognitive abilities on individuals’ educational attainment and labor market outcomes. Concerning educational achievement, non-cognitive abilities independently account for 12% of an individual’s educational attainment (Cunha et al., 2010), with the trait of openness within these abilities being predictive of students’ SAT scores (Noftle and Robins, 2007). Regarding labor market achievements, Cobb-Clark and Tan (2011) analyze data from the Australian Household, Income and Labor Dynamics in Australia (HILDA) Survey to reveal that a one standard deviation increase in agreeableness reduces the likelihood of men becoming managers by 2.8%, while a similar increase in conscientiousness raises this likelihood by 2%. For women, a one standard deviation increase in openness(extraversion) increases their chances of becoming managers by 2.5% (1.2%). Heineck (2011), Nandi and Nicoletti (2014), using data from the British Household Panel survey, independently find that openness measured through the Big Five personality inventory is the primary determinant influencing wage disparities.

This study utilizes data from the China Education Panel Survey and employs multiple linear regression models and propensity score matching to examine the impact of co-residing with grandparents on adolescents’ non-cognitive abilities. Additionally, robustness assessments are conducted using the augmented inverse probability weighting method and placebo tests. This research contributes to existing literature in three key aspects. Firstly, unlike previous studies that often categorize adolescent co-residence with grandparents under grandparent-grandchild co-residence without considering structural differences between three-generation co-residence and skip-generation co-residence, this study distinguishes between these two types of arrangements, allowing for a more nuanced analysis of their effects on adolescents’ non-cognitive abilities. Secondly, while there is an abundance of literature focusing on academic performance and student health, research specifically examining the influence of grandparent-grandchild co-residence on non-cognitive abilities has been relatively limited. Thirdly, in terms of mechanism analysis, this paper investigates the mediating variables that account for changes in adolescents’ non-cognitive abilities. The presence of grandparents in three-generation co-residences enables parents to allocate more attention to child care responsibilities, thereby positively influencing their children’s non-cognitive abilities.

The paper is structured as follows: the second section provides a comprehensive review of relevant literature, the third section outlines the data sources and research design employed in this study, the fourth section presents detailed findings and analysis derived from the research, and finally, the fifth section offers conclusions.

## 2 Literature review

### 2.1 Impact of grandparent-grandchild co-residence on adolescent development

The research topic of family residence structure has garnered significant attention from scholars. Variations in ethnic groups and cultural backgrounds exert influence on the selection of family residence structures (Lee, 2023). Huang et al. (2023) discovered that the intergenerational transmission of grandparents’ influence varies based on diverse family residence structures, with the grandparents’ impact on their grandchildren being conveyed through their cognitive processes and socioeconomic status. The attitude of grandparents can exert a positive influence on co-residing grandchildren. Previous research has primarily focused on examining the impact of grandparental involvement in caregiving on various aspects of adolescent development, such as academic performance and physical health. In terms of academic performance, Pong and Chen (2010) discovered, based on samples from Taiwan, China, that cohabitation with grandparents has a significant positive impact on the academic performance of adolescents. Deng et al. (2023) discovered that children residing in multigenerational households, where grandparents assumed the role of primary caregivers (i.e., three generations cohabiting), exhibited superior cognitive performance on standardized tests. Conversely, no significant disparities in cognitive abilities were observed between children living solely with both parents and those residing exclusively with their grandparents (known as skip-generation co-residence). However, Zeng and Hong (2020) found no significant influence of skip-generation co-residence during the preschool stage on the academic performance and cognitive abilities of grandchildren during their secondary school years. Zhang and Wu (2020), utilizing data from the China Education Panel Survey (CEPS), revealed that adolescents residing in three-generation households exhibited significantly superior academic performance compared to students living with their parents. This effect was mediated by family social capital and socioeconomic status as intermediate mechanisms influencing adolescent academic achievement. Conversely, Geng (2020) conducted an analysis using data from the Chinese Family Tracking Survey in 2014 and identified a noteworthy negative impact of three-generation co-residence on the academic performance of grandchildren. In terms of physical well-being, Man et al. (2019) conducted an analysis using data from the Chinese Family Panel Survey in 2016 to examine the impact of skip-generation care on the health of children aged 0–6. The findings suggested that children who received complete or partial skip-generation care exhibited significantly lower levels of overall health compared to those receiving parental care. Similarly, He et al. (2018), utilizing data from the China Health and Nutrition Survey (CHNS), discovered a significant association between living with grandparents and an increased likelihood of obesity among grandchildren. However, there is a dearth of studies comparing the effects of three-generation co-residence and skip-generation co-residence on non-cognitive abilities in adolescents, particularly when considering the nuanced categorization of family residence structure.

### 2.2 Impact of grandparent-grandchild co-residence on adolescents’ non-cognitive abilities

Non-cognitive abilities are a crucial component of the emerging human capital theory (Duckworth et al., 2019), referring to emotional and behavioral competencies demonstrated during interpersonal interactions (Roberts, 2009). Given its significance as a determinant of family environment, it is imperative to investigate the impact of family residence structure on adolescents’ non-cognitive abilities. Xing and Zhang’s (2020) study utilized data from the Chinese Education Panel Survey to assess students’ non-cognitive abilities across dimensions such as environmental adaptation, emotional regulation, self-regulation, and self-efficacy. Their findings revealed that middle school students who received care from their grandparents exhibited significantly lower levels of non-cognitive abilities compared to those receiving care from their parents. Ao et al. (2022) discovered that grandparents exhibited a comparatively less stringent approach toward their grandchildren in contrast to the parents, and children who received primary care from grandparents demonstrated higher levels of extrinsic control and were more inclined to attribute their achievements to external motivation. Notably, this study focused solely on co-residence with grandparents without explicitly distinguishing between skip-generation co-residence and three-generation co-residence. In contrast, Tian and Zhou’s (2021) analysis of data from the China Education Panel Survey argued that skip-generation care had a significantly positive impact on adolescents’ cognitive abilities but did not exert a significant effect on their non-cognitive abilities. These divergent conclusions highlight the need for further research after refining the classification of family residence structures.

In conclusion, the existing literature has made significant contributions; however, there is still scope for further development. Firstly, previous studies have not adequately differentiated between various residence structures within multi-generational households, such as three-generation co-residence and skip-generation co-residence. This paper proposes a subdivision of grandparent-grandchild co-residence into these two categories to better comprehend the varying degrees of caregiving responsibilities assumed by grandparents toward adolescents. The extent of this substitution would have distinct impacts on adolescent development. Secondly, the current literature on grandparent-grandchild co-residence tends to primarily focus on outcome variables like physical health and academic performance among adolescents while neglecting their non-cognitive abilities. Therefore, this study aims to address this gap by examining the effects of three-generation co-residence and skip-generation co-residence on the non-cognitive abilities of adolescents in comparison to two-parent co-residence.

## 3 Data sources and research design

### 3.1 Data sources

The data utilized in this paper is derived from the baseline data (2013–2014) of the China Education Panel Survey (CEPS), a project designed and implemented by the National Survey Research Center (NSRC) at Renmin University of China. The survey specifically targets seventh and ninth-grade students in 28 randomly selected counties nationwide. With a sample size of approximately 20,000 students from 112 schools and 438 classes, this dataset serves as a representative resource for investigating the impact of various factors on students’ cognitive and non-cognitive abilities (Gong et al., 2021; Huang et al., 2021; Abbasi et al., 2022).

The distribution of four types of family residence structures is presented in Table 2. Among the surveyed students, 56.68% live solely with their parents, while 19.25% reside in three-generation households. Skip-generation households account for 6.84%, and other residence structures make up 17.24%. To address the research questions, this study excludes alternative residence structures and eliminates samples with missing values, resulting in a total of 13,183 included samples for analysis. Of these, there are 8,934 samples (67.76%) from two-parent households, 3,192 samples (24.21%) from three-generation households, and 1,057 samples (8.03%) from skip-generation households.

**Table 2:** Description of the distribution of family residential structures.

	Two-parent co-residence families	Three-generation co-residence families	Skip-generation co-residence families	Other residence structures
Sample size	11,045	3,751	1,333	3,358
Proportion (%)	56.68	19.25	6.84	17.24

### 3.2 Key variables

#### 3.2.1 Dependent variable

The non-cognitive abilities of adolescents serve as the dependent variable in this study. However, the CEPS survey lacks a dedicated measurement tool for assessing these abilities. To address this limitation, we adopt assessments of the Big Five personality traits and relevant questions from CEPS to establish three variables that effectively evaluate non-cognitive abilities: *emotional stability, conscientiousness*, and *agreeableness*. This approach builds upon previous studies (e.g., Sorić et al., 2017; Pang et al., 2020; Zheng et al., 2022) with similar objectives. Detailed descriptions of the measurement methods employed for these three variables can be found in Table 3.

**Table 3:** Non-cognitive abilities measures.

Non-cognitive abilities	Corresponding questions in the CEPS questionnaire	Original options
Emotional stability	Feeling down	Likert 5-level scoring method, 1 = never, 2 = rarely, 3 = sometimes, 4 = often, 5 = always, with higher numbers representing a higher frequency of the corresponding emotion.
	Feeling depressed	
	Feeling unhappy	
	Feeling life is meaningless	
	Feeling sad	
Conscientiousness	Even when feeling slightly uncomfortable or facing other obstacles, I consistently strive to attend school.	Likert 4-level scoring method, 1 = completely disagree, 2 = somewhat disagree, 3 = somewhat agree, 4 = completely agree
	Regardless of my dislike for a particular subject, I exert maximum effort in completing assigned tasks.	
	Despite the time-consuming nature of homework, I persistently endeavor to complete it.	
Agreeableness	Most classmates exhibit friendliness toward me in the class.	Likert 4-level scoring method, 1 = completely disagree, 2 = somewhat disagree, 3 = somewhat agree, 4 = completely agree
	The classroom environment is conducive to learning.	
	I actively participate in school or class activities.	
	I feel a sense of closeness with my peers at this school.	

##### 3.2.1.1 Emotional stability

This study utilizes data from the CEPS survey, specifically focusing on responses to the question “In the past seven days, have you experienced any of the following emotions?” The five items included in this analysis are “feeling down” “feeling depressed” “feeling unhappy” “feeling life is meaningless” and “feeling sad.” Initially, reverse scoring was applied to these items, with higher values indicating lower levels of emotional stability among students. Subsequently, a factor analysis was conducted on these five items, revealing that they can be effectively summarized by a single factor termed as “emotional stability.” The variable value represents the corresponding factor score for each individual student, where higher values indicate greater non-cognitive abilities within this dimension for adolescents.

##### 3.2.1.2 Conscientiousness

This study utilizes data from the CEPS survey, specifically focusing on responses to three items regarding self-perception during sixth grade: “Even when feeling slightly uncomfortable or facing other obstacles, I consistently strive to attend school,” “Regardless of my dislike for a particular subject, I exert maximum effort in completing assigned tasks,” and “Despite the time-consuming nature of homework, I persistently endeavor to complete it.” Factor analysis is employed to examine these items, revealing that they can be effectively summarized by a single factor termed “conscientiousness.” The variable value corresponds to the factor score, with higher values indicating greater non-cognitive abilities within this dimension among adolescents.

##### 3.2.1.3 Agreeableness

This study utilizes CEPS data on students’ perceptions of school life, specifically focusing on four items: “Most classmates exhibit friendliness towards me in the class,” “The classroom environment is conducive to learning,” “I actively participate in school or class activities,” and “I feel a sense of closeness with my peers at this school.” A factor analysis was conducted on these items, revealing that they can be consolidated into one factor termed as “agreeableness.” The variable value represents the factor score, where higher values indicate greater non-cognitive abilities within this dimension for adolescents.

#### 3.2.2 Independent variable

The primary independent variable examined in this study is the co-residence situation between grandparents and grandchildren. Based on data from the CEPS survey, family living arrangements were categorized into three groups according to responses to the question “Who do you currently live with in your home?”: two-parent co-residence (adolescents living solely with their parents), three-generation co-residence (adolescents living with both parents and grandparents), and skip-generation co-residence (adolescents living solely with their grandparents). Among these categories, two-parent co-residence is considered the most advantageous family structure or living arrangement for children’s development (Wu et al., 2018) and serves as the control group in our empirical analysis. The impact of three-generation co-residence on the non-cognitive abilities of adolescents is measured through variable $\mathrm{Thre}e_{i}$ , where $\mathrm{Thre}e_{i}$ equals 1 represents three-generation co-residence, and $\mathrm{Thre}e_{i}$ equals 0 represents skip-generation co-residence or two-parent co-residence. The impact of skip-generation co-residence on the non-cognitive abilities of adolescents is measured through variable $\mathrm{Ski}p_{i}$ , where $\mathrm{Ski}p_{i}$ equals 1 represents skip-generation co-residence, and $\mathrm{Ski}p_{i}$ equals 0 represents three-generation co-residence or two-parent co-residence.

#### 3.2.3 Control variables

This paper draws on previous literature in setting control variables (Gong et al., 2021). Gender (1 = female, 0 = male), age (calculated by subtracting the year of birth from the survey year), minority status (1 = yes, 0 = no/Han ethnicity), only-child status (1 = yes, 0 = no), rural household registration (1 = yes, 0 = no), preschool attendance status (1 = yes, 0 = no), migrant children (1 = yes, 0 = no), and boarding status (1 = yes, 0 = no) are treated as control variables in this paper.

### 3.3 Model specification and analysis procedures

#### 3.3.1 Multiple linear regression models

To estimate the impact of grandparent-grandchild co-residence on non-cognitive abilities in adolescents, this paper first employs a multiple linear regression model for estimation. The model is as follows:


(1) $$Y_{i}=\beta_{0}+\beta_{1}\mathrm{Thre}e_{i}+\beta_{2}\mathrm{Ski}p_{i}+\sum \gamma X_{i}+\varepsilon_{i}$$

$Y_{i}$ represents the non-cognitive abilities of adolescents. $\mathrm{Thre}e_{i}$ represents whether the adolescents is from a three-generation co-residence (yes = 1, no = 0); $\mathrm{Ski}p_{i}$ represents whether the adolescents is from a skip-generation co-residence (yes = 1, no = 0); Two-parent co-residence (living only with both father and mother) serves as the control group; $X_{i}$ represents a series of individual characteristics, including age, minority, gender, household registration, preschool attendance, whether the student is a migrant child, whether they are only-child, and whether they board in school; $\varepsilon_{i}$ represents individual-level random error term. The coefficients of interest in this paper are $\beta_{1}$ and $\beta_{2}$ , which, respectively, measure the differences in non-cognitive abilities between adolescents from three-generation, skip-generation co-residence and those two-parent co-residence households.

#### 3.3.2 Propensity score matching method

The utilization of ordinary least squares (OLS) estimation in Equation (1) may fail to capture the treatment effect of grandparent-grandchild co-residence on the non-cognitive abilities of adolescents, which is a key focus for researchers. Propensity score matching estimators are commonly employed in academic papers to estimate both average treatment effects and the average treatment effect on the treated (Abadie and Imbens, 2016). Given that both family environment and individual characteristics of adolescents can influence both the residence structure within families and the development of grandchildren, it results in non-randomness in the residence structure and introduces endogeneity issues due to sample selection bias. Furthermore, within the framework of counterfactual analysis, OLS estimation solely provides an assessment of the average treatment effect (ATE) associated with grandparent co-residence; this measures differences in development between three-generation (or skip-generation) co-residence and two-parent co-residence for adolescents. If we denote $GG_{i}$ as the dummy variable representing whether grandchild i live with grandparents or not, $Y_{0i}$ representing non-cognitive abilities of adolescent i who is residence with two parents only, $Y_{1i}$ representing non-cognitive abilities of grandchild i who is in three-generation (or skip-generation) co-residence family, the average treatment effect can be represented by Equation (2):


(2) $$\mathrm{ATE}=E\left(Y_{1i}-Y_{0i}|X\right)=E\left(Y_{1i}|GG_{i}=1,X\right)-E\left(Y_{0i}|GG_{i}=0,X\right)$$

However, researchers are primarily interested in comparing the developmental levels of adolescents who have experienced three-generation (or skip-generation) co-residence with those who have not, in order to examine potential differences.


(3) $$E\left(Y_{1i}-Y_{0i}|,ZS_{i}=1|,X\right)=E\left(Y_{1i}|ZS_{i}=1,X\right)-E\left(Y_{0i}|ZS_{i}=1,X\right)$$

Researchers are also interested in comparing the developmental levels of students residing in two-parent co-residence households with those of students residing in three-generation (or skip-generation) co-residence households.


(4) $$E\left(Y_{1i}-Y_{0i}|,ZS_{i}=0|,X\right)=E\left(Y_{1i}|ZS_{i}=0,X\right)-E\left(Y_{0i}|ZS_{i}=0,X\right)$$

Equations (3) and (4) are referred to as the Average Treatment Effect on Treated (ATT) and Average Treatment Effect on Untreated (ATU), respectively. This paper primarily focuses on the ATT.

#### 3.3.3 Augmented inverse probability weighting method

In order to mitigate the potential impact of family selection bias on estimation results, this study also employs the augmented inverse probability weighting method. Inverse probability weighting (IPW) is a widely used approach for adjusting unequal sampling fractions in sample surveys (Seaman and White, 2013). The augmented inverse probability weighting (AIPW) method aims to enhance the limitations of IPW and provide a more robust approach for estimating causal effects. AIPW integrates the concepts of inverse probability weighting and outcome regression estimation, thus commonly referred to as a doubly robust methodology. This implies that even if one of the propensity score models or outcome regression models is mis-specified, AIPW can still yield unbiased estimates of causal effects (given that the other model is accurate). This dual robustness represents a significant advantage of AIPW over IPW, rendering it more reliable and resilient in practical applications. The specific operational steps of this method are as follows: Firstly, calculate the probabilities of adolescents entering different family residence structures. Subsequently, compute weights based on these probabilities to construct a weighted sample that balances the sizes of different family residence structures and control variables. The formula for calculating weights associated with entering various family residence structures is as follows in Equation (5):


(5) $${CW}_{i}=\frac{P\left(S_{i}=s_{i}\right)}{P\left(S_{i}=s_{i}|C_{i}\right)}$$

${CW}_{i}$ represents the weights for adolescents entering different family residence structures. $S_{i}$ represents different family residence structures. $C_{i}$ represents the control variables that influence adolescents’ entry into different family residence structures. These variables include the age, gender, minority, agricultural household registration, preschool attendance, migrant children, only-children, boarding status, and the parents’ highest level of education.

## 4 Results

### 4.1 Descriptive statistics

Table 4 presents the descriptive statistics of key demographic characteristics among adolescents in the sample. Non-cognitive abilities are standardized with a mean of 0 and a standard deviation of 1. The average age of adolescents in the sample is 14.49 years, with approximately 8% representing students from ethnic minorities and 92% being Han Chinese students. The proportion of female students is 51%, while male students account for 49%. The sample includes an equal distribution between students from agricultural and non-agricultural households, each comprising 50%. Around 81% of the students have attended preschool, while the remaining 19% have not. Amongst the student population, 18% come from migrant families, whereas non-migrants make up the remaining 82%. Only-child students constitute 45%, while non-only-child students account for 55%. Boarding school attendees represent 30%, while non-boarding school attendees comprise 70%. On average, parents’ educational attainment is 10.92 years, which corresponds to completion of junior high school.

**Table 4:** Description of basic characteristics of adolescents (*N* = 13,183).

Variables	Mean	Std. dev	Min	Max
**Non-cognitive abilities (dependent variables)**				
Emotional stability	0.0117	0.985	−3.559	1.310
Agreeableness	0.0318	0.977	−3.125	1.356
Conscientiousness	0.0291	0.983	−3.468	1.016
**Family residence structure (independent variables)**				
Two-parent co-residence	0.6776	0.467	0	1
Three-generation co-residence	0.2421	0.428	0	1
Skip-generation co-residence	0.0803	0.272	0	1
**Control variables**				
Age	14.49	1.238	12	18
Minority	0.0771	0.267	0	1
Gender	0.512	0.500	0	1
Rural household registration	0.501	0.500	0	1
Preschool attendance	0.815	0.388	0	1
Migrant children	0.182	0.386	0	1
Only-child	0.454	0.498	0	1
Boarding	0.303	0.460	0	1
Parents’ highest level of education	10.92	3.031	0	19

The characteristics of adolescents among different family residence structures are compared in Table 5. Columns 1, 2, and 3 present the mean and standard deviation of non-cognitive abilities and relevant demographic characteristics of adolescents from households with two-parent co-residence, three-generation co-residence, and skip-generation co-residence, respectively. Columns 4, 5, and 6 display the t-tests for mean differences in non-cognitive abilities and relevant demographic characteristics between two-parent co-residence and three-generation co-residence, two-parent co-residence and skip-generation co-residence, as well as three-generation co-residence and skip-generation co-residence.

**Table 5:** Differences in the characteristics of adolescents in different family residence structures.

	Two-parent co-residence	Three-generation co-residence	Skip-generation co-residence	Mean comparison test	Mean comparison test	Mean comparison test
	(1)	(2)	(3)	(4)	(5)	(6)
Variables	Mean	Mean	Mean	(1)–(2)	(1)–(3)	(2)–(3)
Emotional stability	0.012	0.078	−0.194	−0.066***	0.206***	0.272***
	(0.995)	(0.945)	(0.990)			
Agreeableness	0.028	0.111	−0.177	−0.082***	0.206***	0.288***
	(0.981)	(0.956)	(0.972)			
Conscientiousness	0.008	0.085	0.040	−0.078***	−0.032	0.046
	(0.996)	(0.966)	(0.917)			
Age	14.515	14.376	14.627	0.139***	−0.112***	−0.252***
	(1.233)	(1.206)	(1.338)			
Minority	0.077	0.067	0.106	0.011*	−0.029***	−0.039***
	(0.267)	(0.250)	(0.308)			
Gender	0.505	0.537	0.489	−0.031***	0.016	0.048***
	(0.500)	(0.499)	(0.500)			
Rural household registration	0.479	0.502	0.687	−0.022**	−0.208***	−0.185***
	(0.500)	(0.500)	(0.464)			
Preschool attendance	0.814	0.841	0.742	−0.026***	0.073***	0.099***
	(0.389)	(0.366)	(0.438)			
Migrant children	0.220	0.113	0.076	0.107***	0.144***	0.037***
	(0.414)	(0.317)	(0.265)			
Only-child	0.456	0.516	0.243	−0.059***	0.213***	0.273***
	(0.498)	(0.500)	(0.429)			
Boarding	0.263	0.320	0.592	−0.057***	−0.329***	−0.273***
	(0.440)	(0.466)	(0.492)			
Parents’ highest level of education	10.982	11.150	9.703	−0.168***	1.279***	1.447***
	(3.076)	(2.980)	(2.473)			
Observations	8,934	3,192	1,057			

*p < 0.1, **p < 0.05, ***p < 0.01, Standard errors are shown in parentheses.

The results of mean comparison tests for non-cognitive abilities reveal that adolescents residing in three-generation households exhibit significantly higher levels of emotional stability compared to those living in two-parent households and skip-generation households. Moreover, adolescents in three-generation households demonstrate significantly greater agreeableness than their counterparts in two-parent households and skip-generation households. Additionally, adolescents in three-generation households display notably elevated conscientiousness when compared to those in two-parent households, while no significant difference is observed between adolescents in two-parent households and those in skip-generation households regarding conscientiousness.

The results of the mean comparison test for individual characteristics indicate that adolescents residing in three-generation households exhibit significantly lower ages compared to those living in two-parent households, while skip-generation households demonstrate significantly higher ages than two-parent households. Moreover, the proportion of ethnic minority adolescents is higher in two-parent households than in three-generation households, and it is even higher in skip-generation households compared to two-parent ones. Additionally, the percentage of female students is lower in two-parent households than in three-generation ones, with the lowest proportion observed among skip-generation households. Furthermore, three-generation households have a smaller representation of individuals with rural household registration, whereas skip-generation homes possess the highest proportion. In terms of preschool attendance, the proportion of adolescents receiving preschool education is higher in three-generation households compared to two-parent households, while it is higher in two-parent households than skip-generation households. Regarding being an only-child, three-generation households exhibit the highest proportion of single children, whereas skip-generation households have the lowest proportion. Concerning boarding school enrollment, skip-generation households demonstrate the highest proportion of adolescents attending boarding schools. With regards to parental education levels, three-generation households exhibit significantly higher levels of education among parents compared to both two-parent and skip-generation households.

### 4.2 Regression results

#### 4.2.1 Multivariate linear regression results

The results of the multivariate linear regression model, which estimates the impact of co-residence with grandparents on non-cognitive abilities in adolescents, are presented in Table 6. Table 6 exhibits the regression outcomes for three dimensions of non-cognitive abilities influenced by co-residence with grandparents. Columns 1, 3, and 5 represent the estimates of the baseline model, indicating that compared to adolescents in two-parent co-residence households, three-generation co-residence has a significant positive effect on emotional stability, agreeableness, and conscientiousness. However, skip-generation co-residence negatively impacts emotional stability and agreeableness significantly but does not have a significant effect on conscientiousness. Columns 2, 4, and 6 include control variables based on the previous baseline model while maintaining robust estimation results. Specifically, when compared to adolescents living in two-parent co-residence households, those residing with three generations experience a significantly positive effect on emotional stability (0.049 standard deviations), agreeableness (0.063 standard deviations), and conscientiousness (0.045 standard deviations). Conversely, adolescents living in skip-generation households exhibit significantly negative effects on emotional stability (−0.182 standard deviations) and agreeableness (−0.107 standard deviations) compared to those living in two-parent co-residence households.

**Table 6:** Effects of living with grandparents on children’s non-cognitive abilities.

Variables	Emotional stability		Agreeableness		Conscientiousness	
	(1)	(2)	(3)	(4)	(5)	(6)
Three-generation co-residence	0.066***	0.049**	0.082***	0.063***	0.078***	0.045**
	(0.020)	(0.020)	(0.020)	(0.020)	(0.020)	(0.020)
Skip-generation co-residence	−0.206***	−0.182***	−0.206***	−0.107***	0.032	0.007
	(0.032)	(0.033)	(0.032)	(0.032)	(0.032)	(0.032)
Age		−0.092***		−0.029***		−0.119***
		(0.007)		(0.007)		(0.007)
Minority		−0.048		−0.168***		0.039
		(0.032)		(0.032)		(0.032)
Gender		−0.077***		0.153***		0.224***
		(0.017)		(0.017)		(0.017)
Rural household registration		0.059***		−0.003		0.075***
		(0.020)		(0.020)		(0.020)
Preschool attendance		0.080***		0.137***		−0.023
		(0.022)		(0.022)		(0.022)
Migrant children		−0.017		0.024		−0.079***
		(0.023)		(0.022)		(0.023)
Only-child		0.016		0.084***		0.005
		(0.020)		(0.019)		(0.020)
Boarding		−0.025		−0.049**		0.011
		(0.021)		(0.021)		(0.021)
Parents’ highest level of education		0.008**		0.031***		−0.008**
		(0.003)		(0.003)		(0.003)
Constants	0.012	1.216***	0.028***	−0.098	0.008	1.697***
	(0.010)	(0.117)	(0.010)	(0.115)	(0.010)	(0.116)
						
Observations	13,183	13,183	13,183	13,183	13,183	13,183
Adjusted R-squared	0.004	0.023	0.005	0.040	0.001	0.037
$P\left(\beta_{1}=\beta_{2}\right)$	0.000	0.000	0.000	0.000	0.191	0.270

*p < 0.1, **p < 0.05, ***p < 0.01, ****Standard errors are shown in parentheses.

To investigate potential disparities in the influence of various family residence structures on the non-cognitive abilities of adolescents, this study employs t-tests to examine the primary coefficients presented in columns 2, 4, and 6 of Table 6. Our findings reveal noteworthy dissimilarities in the coefficients associated with skip-generation co-residence and three-generation co-residence concerning their impact on emotional stability and agreeableness among adolescents. However, no significant distinction is observed between skip-generation co-residence and three-generation co-residence when analyzing conscientiousness.

In terms of individual characteristics, female students have poorer emotional stability but higher scores in agreeableness and conscientiousness. Students with a rural household registration demonstrate better emotional stability and conscientiousness compared to students with an urban household registration, although their agreeableness is lower. Students who have received preschool education exhibit better emotional stability and agreeableness than those who have not. Migrant children have significantly lower levels of conscientiousness compared to local children, and boarders have lower agreeableness compared to non-boarders.

The findings presented in this paper are consistent with previous literature. For instance, our study aligns with Han et al. (2020) by demonstrating that three-generation co-residence positively influences the non-cognitive development of adolescents compared to two-generation co-residence. Han et al.’s (2020) research indicates that children living with their grandparents exhibit fewer external and internalized behavioral problems than their peers, which is congruent with intergenerational solidarity theory and situational models of family stress. Additionally, Liang (2021) found that three-generation living arrangements effectively enhance family social capital, thereby promoting the development of adolescents’ non-cognitive abilities. Consequently, it is imperative to acknowledge the beneficial impact of multi-generational cohabitation on teenagers’ human capital accumulation. Conversely, the present study reveals a detrimental impact of skip-generation co-residence on adolescents’ non-cognitive abilities. This finding is consistent with existing literature; for instance, Xing and Zhang (2020) demonstrate that skip-generation parenting significantly diminishes the non-cognitive skills of middle school students. Specifically, those who receive skip-generation parenting exhibit lower levels of environmental adaptability, emotional regulation, self-regulation, and self-efficacy perception compared to their counterparts receiving parental care. Moreover, the influence on environmental adaptability and self-efficacy perception is particularly pronounced. Similarly, recent research by Du et al. (2023) indicates that children from disadvantaged families who experience skip-generation parenting display reduced resilience levels. To foster the healthy development of children from challenging backgrounds, it is imperative to implement measures aimed at enhancing grandparents’ attentiveness and caregiving capabilities concerning children’s academic pursuits and daily lives. These efforts will help mitigate the adverse effects of familial difficulties on children’s resilience while ensuring their overall well-being.

#### 4.2.2 Propensity score matching and regression results

In the descriptive statistical analysis, significant differences are observed in personal characteristics such as age, gender, and minority status among various family residence structures. To mitigate the influence of these factors, we employ the propensity score matching method to accurately estimate the impact of family residence structure on non-cognitive abilities of adolescents. The assumptions of independence and common support serve as prerequisites for conducting propensity score matching analysis; hence it is imperative to validate these assumptions prior to applying this method.

##### 4.2.2.1 Balancing assumption

During the analysis, we employed two-parent co-residence as the control group and separately matched skip-generation co-residence and three-generation co-residence to it. The matching process partially addresses endogeneity issues arising from self-selection in family residence structures. As presented in Tables 7–9, significant differences exist in the control variables between three-generation co-residence (skip-generation co-residence) and two-parent co-residence among adolescents before matching (*p*-value = 0.000). However, after matching, no significant differences are observed in the control variables between the three-generation co-residence (skip-generation co-residence) and two-parent co-residence groups (*p*-value >0.1). Furthermore, post-matching results demonstrate a substantial decrease in Pseudo *R*^2^ compared to pre-matching values, with non-significant LR statistics. These findings indicate that the distribution of control variables between the three-generation co-residence (skip-generation co-residence) and two-parent co-residences groups is consistent and satisfies balance test assumptions.

**Table 7:** Near-neighbor matching balance test.

	Variable	Matched or not	Three-generation vs two-parent co-residence					Skip-generation vs two-parent co-residence				
			Three-generation	Two-parent co-residence	Bias (%)	*T*-value	*p* > |*t*|	Skip-generation	Two-parent co-residence	Bias (%)	Two-parent co-residence	*p* > |*t*|
(1)	Age	Unmatched	14.376	14.515	−11.400	−5.53	0.000	14.627	14.515	8.70	2.77	0.006
		Matched	14.376	14.363	1	0.410	0.685	14.625	14.577	3.7	0.84	0.402
(2)	Minority	Unmatched	0.067	0.077	−4.100	−1.960	0.050	0.106	0.077	9.9	3.24	0.001
		Matched	0.067	0.052	5.9	2.57	0.010	0.105	0.093	4.1	0.92	0.359
(3)	Gender	Unmatched	0.537	0.505	6.3	3.060	0.002	0.489	0.505	−3.3	−1.01	0.314
		Matched	0.537	0.533	0.7	0.30	0767	0.490	0.483	1.2	0.29	0.774
(4)	Rural household registration	Unmatched	0.502	0.479	4.5	2.160	0.031	0.687	0.479	43.1	12.87	0.000
		Matched	0.502	0.502	−0.100	−0.02	0.984	0.687	0.688	−0.4	−0.09	0.925
(5)	Preschool attendance	Unmatched	0.841	0.814	7	3.350	0.001	0.742	0.814	−17.6	−5.67	0.000
		Matched	0.841	0.848	−1.900	−0.79	0.427	0.742	0.754	−2.8	−0.61	0.541
(6)	Migrant children	Unmatched	0.113	0.220	−29.000	−13.240	0.000	0.076	0.220	−41.5	−11.05	0.000
		Matched	0.113	0.112	0.3	0.15	0.880	0.076	0.072	1.1	0.35	0.727
(7)	Only-child	Unmatched	0.516	0.456	11.900	5.760	0.000	0.243	0.456	−45.9	−13.35	0.000
		Matched	0.516	0.512	0.8	0.30	0.764	0.243	0.238	1.1	0.27	0.784
(8)	Boarding	Unmatched	0.320	0.263	12.500	6.130	0.000	0.592	0.263	70.5	22.69	0.000
		Matched	0.320	0.312	1.7	0.67	0.504	0.592	0.582	2.1	0.45	0.652
(9)	Parents’ highest level of education	Unmatched	11.150	10.982	5.5	2.67	0.008	9.703	10.982	−45.8	−13.03	0.000
		Matched	11.150	11.118	1.1	0.43	0.667	9.712	9.699	0.5	0.12	0.901
Sample				Pseudo *R*^2^	LRchi^2^	*p* > chi^2^			Pseudo *R*^2^	LRchi^2^	*p* > chi^2^	
Unmatched				0.023	319.52	0.000			0.098	662.080	0.000	
Matched				0.001	9.03	0.435			0.001	2.380	0.984	

**Table 8:** Radius matching balance test.

	Variable	Matched or not	Three-generation vs two-parent co-residence					Skip-generation vs two-parent co-residence				
			Three-generation	two-parent co-residence	Bias (%)	*T*-value	*p* > |*t*|	Skip-generation	two-parent co-residence	Bias (%)	Two-parent co-residence	*p* > |*t*|
(1)	Age	Unmatched	14.376	14.515	−11.4	−5.52	0.000	14.627	14.515	8.7	2.77	0.006
		Matched	14.376	14.383	−0.6	−0.26	0.797	14.627	14.644	−1.3	−0.30	0.764
(2)	Minority	Unmatched	0.067	0.077	−4.1	−1.96	0.050	0.106	0.077	9.9	3.24	0.001
		Matched	0.067	0.067	0.0	0.00	0.996	0.104	0.106	−0.800	−0.16	0.871
(3)	Gender	Unmatched	0.537	0.505	6.3	3.06	0.222	0.489	0.505	−3.300	−1.01	0.314
		Matched	0.537	0.535	0.4	0.16	0.871	0.490	0.491	−0.200	−0.04	0.967
(4)	Rural household registration	Unmatched	0.502	0.479	4.5	2.16	0.031	0.687	0.479	43.100	12.87	0.000
		Matched	0.502	0.503	−0.3	−0.10	0.917	0.686	0.687	−0.400	−0.08	0.933
(5)	Preschool attendance	Unmatched	0.841	0.814	7.0	3.35	0.001	0.742	0.814	−17.600	−5.67	0.000
		Matched	0.841	0.841	−0.2	−0.07	0.946	0.744	0.740	0.8	0.17	0.864
(6)	Migrant children	Unmatched	0.113	0.220	−29.0	−13.24	0.000	0.076	0.220	−41.500	−11.05	0.000
		Matched	0.113	0.114	−0.3	−0.13	0.896	0.076	0.079	−1.000	−0.29	0.773
(7)	Only-child	Unmatched	0.516	0.456	11.9	5.76	0.000	0.243	0.456	−45.900	−13.35	0.000
		Matched	0.516	0.513	0.5	0.21	0.836	0.244	0.243	0.2	0.06	0.953
(8)	Boarding	Unmatched	0.320	0.263	12.5	6.13	0.000	0.592	0.263	70.500	22.69	0.000
		Matched	0.320	0.324	−1.0	−0.38	0.704	0.591	0.591	0.000	−0.01	0.995
(9)	Parents’ highest level of education	Unmatched	11.150	10.982	5.5	2.67	0.000	9.703	10.982	−45.800	−13.03	0.000
		Matched	11.150	11.158	−0.3	−0.10	0.918	9.728	9.689	1.4	0.36	0.715
Sample				Pseudo *R*^2^	LRchi^2^	*p* > chi^2^			Pseudo *R*^2^	LRchi^2^	*p* > chi^2^	
Unmatched				0.023	319.520	0.000			0.098	662.08	0.000	
Matched				0.000	0.370	1.000			0.000	0.320	1.000	

**Table 9:** Kernel matching balance test.

	Variable	Matched or not	Three-generation vs two-parent co-residence					Skip-generation vs two-parent co-residence				
			Three-generation	two-parent co-residence	Bias (%)	*T*-value	*p* > |*t*|	Skip-generation	two-parent co-residence	Bias (%)	Two-parent co-residence	*p* > |*t*|
(1)	Age	Unmatched	14.376	14.515	−11.400	−5.52	0.000	14.627	14.515	2.770	2.77	0.006
		Matched	14.376	14.375	0.1	0.02	0.981	14.627	14.638	90.100	−0.19	0.846
(2)	Minority	Unmatched	0.067	0.077	−4.100	−1.96	0.050	0.106	0.077	3.240	3.24	0.001
		Matched	0.067	0.066	0.1	0.04	0.968	0.104	0.105	93.600	−0.14	0.890
(3)	Gender	Unmatched	0.537	0.505	6.3	3.06	0.002	0.489	0.505	−1.010	−1.01	0.314
		Matched	0.537	0.538	−0.300	−0.11	0.911	0.490	0.493	83.100	−0.13	0.899
(4)	Rural household registration	Unmatched	0.502	0.479	4.5	2.16	0.031	0.687	0.479	12.870	12.87	0.000
		Matched	0.502	0.499	0.5	0.19	0.852	0.686	0.689	98.500	−0.15	0.880
(5)	Preschool attendance	Unmatched	0.841	0.814	7	3.35	0.001	0.742	0.814	−5.670	−5.67	0.000
		Matched	0.841	0.842	−0.400	−0.17	0.866	0.744	0.740	94.700	0.20	0.839
(6)	Migrant children	Unmatched	0.113	0.220	−29.000	−13.24	0.000	0.076	0.220	−11.050	−11.05	0.000
		Matched	0.113	0.113	−0.100	−0.03	0.975	0.076	0.079	98.100	−0.24	0.810
(7)	Only-child	Unmatched	0.516	0.456	11.900	5.76	0.000	0.243	0.456	−13.350	−13.35	0.000
		Matched	0.516	0.517	−0.300	−0.12	0.904	0.244	0.245	99.500	−0.05	0.958
(8)	Boarding	Unmatched	0.320	0.263	12.500	6.13	0.000	0.592	0.263	22.690	22.69	0.000
		Matched	0.320	0.316	0.9	0.34	0.736	0.591	0.591	100.000	−0.01	0.996
(9)	Parents’ highest level of education	Unmatched	11.150	10.982	5.5	2.67	0.008	9.703	10.982	−13.030	−13.03	0.000
		Matched	11.150	11.177	−0.900	−0.36	0.720	9.728	9.686	96.700	0.40	0.693
Sample				Pseudo *R*^2^	LRchi^2^	*p* > chi^2^			Pseudo *R*^2^	LRchi^2^	*p* > chi^2^	
Unmatched				0.023	319.52	0.000			0.098	662.08	0.000	
Matched				0.000	0.22	1.000			0.000	0.33	1	

##### 4.2.2.2 Common support assumption

**Figure 1:**
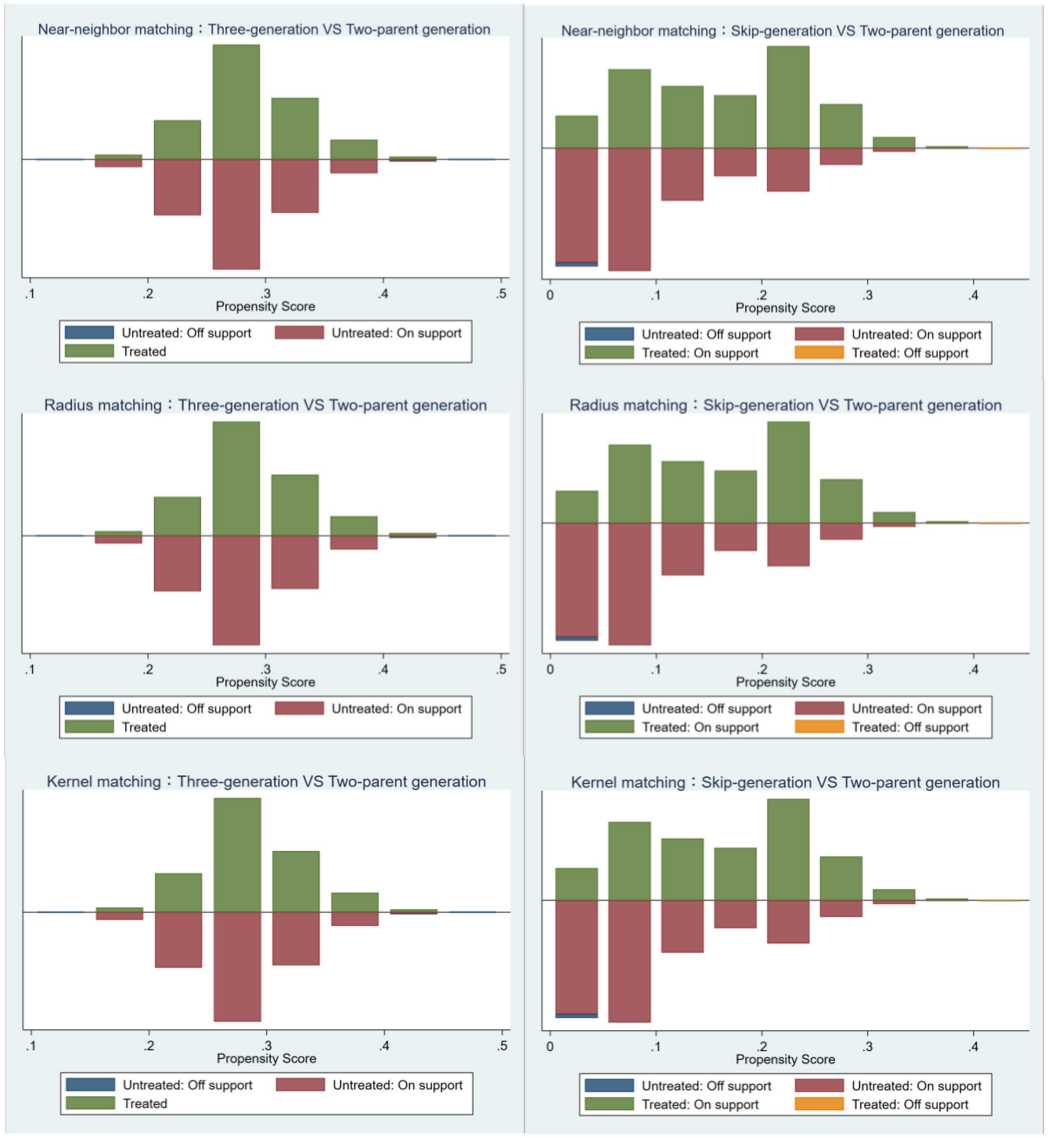
Comparison of propensity scores for different matching components.

Before estimating the treatment effects of grandparent-grandchild co-residence on the non-cognitive abilities of adolescents, it is essential to conduct a hypothesis test for common support on the matched sample. As depicted in Figure 1, it is evident that only a minimal number of samples fall outside the common support region, and the propensity scores exhibit an adequately large common support region in both three-generation (skip-generation) co-residence and two-parent co-residence samples, thereby satisfying the assumption of common support.

##### 4.2.2.3 Regression results after propensity score matching

Based on the above analysis, it is evident that the matched samples fulfill the prerequisites for employing propensity score matching. This study employs nearest neighbor matching, radius matching, and kernel matching techniques based on the baseline model to compute average treatment effects (ATE) on adolescents’ non-cognitive abilities resulting from three-generation and skip-generation co-residence. The outcomes presented in Table 10 reveal that three-generation co-residence significantly enhances emotional stability, agreeableness, and conscientiousness among adolescents in terms of their non-cognitive abilities. Conversely, skip-generation co-residence exhibits a significant negative impact on emotional stability and agreeableness among adolescents. The Average Treatment Effect on the Treated (ATT) for the influence of three-generation co-residence on emotional stability amounts to 0.049 and 0.048 using radius matching and kernel matching methods respectively; both statistically significant at a significance level of 5%. Similarly, ATT for the effect of three-generation co-residence on agreeableness stands at 0.062 and 0.058 with radius matching and kernel matching respectively; both statistically significant at a significance level of 1%. Lastly, ATT for the impact of three-generation co-residence on conscientiousness equals to 0.048 with radius matching method while it is slightly lower at 0.047 when utilizing kernel matching technique; both statistically significant at a significance level of 5%.

**Table 10:** Effects of living with grandparents on children’s non-cognitive abilities estimates by PSM.

Control group: two-parent co-residence	Matching method	Emotional stability			Agreeableness			Conscientiousness		
		ATT	SE (standard error)	*T*-value	ATT	SE (standard error)	*T*-value	ATT	SE (standard error)	*T*-value
Three-generation co-residence	Near-neighbor matching	−0.044	0.034	−1.28	−0.077	0.034	−2.26**	−0.066	0.035	−1.90**
	Radius matching	0.049	0.020	2.44**	0.062	0.020	3.05***	0.048	0.020	2.36**
	Kernel matching	0.048	0.020	2.38**	0.058	0.020	2.88***	0.047	0.020	2.31**
Skip-generation co-residence	Near-neighbor matching	−0.23	0.045	−5.14***	−0.241	0.046	−5.30***	−0.075	0.043	−1.74*
	Radius matching	−0.181	0.034	−5.38***	−0.099	0.033	−3.00***	0.020	0.032	0.63
	Kernel matching	−0.184	0.034	−5.46***	−0.010	0.033	−3.01***	0.021	0.032	0.67

*p < 0.1, **p < 0.05, ***p < 0.01.

The ATT for the impact of skip-generation co-residence on adolescents’ emotional stability is −0.23, −0.181, and −0.184 for nearest neighbor matching, radius matching, and kernel matching, respectively, all statistically significant at the 1% level. Similarly, the ATT for the impact of skip-generation co-residence on adolescents’ agreeableness is −0.241, −0.099, and − 0.010 for nearest neighbor matching, radius matching, and kernel matching, respectively, all statistically significant at the 1% level. However, no significant impact of skip-generation co-residence was observed on adolescents’ conscientiousness.

The findings derive from employing both ordinary least squares and propensity score matching approaches reveal that a significant positive impact of three-generation co-residence on the non-cognitive abilities of adolescents. In contrast, skip-generation co-residence is found to have an adverse effect on adolescents’ non-cognitive abilities.

### 4.3 Robustness check

This paper employs the augmented inverse propensity weighting method and placebo test to validate the robustness of the results.

#### 4.3.1 Augmented inverse probability weighting (AIPW) method

In this section, we employ the augmented inverse probability weighting (AIPW) method to assess the robustness of our findings. The two-parent co-residence will serve as the reference group, while comparisons will be made with both three-generation co-residence and skip-generation co-residence. To estimate individual probabilities of entering a three-generation or skip-generation household, logistic regression modeling is utilized. Subsequently, a weighted sample is constructed based on these probabilities to achieve data balance.

**Figure 2:**
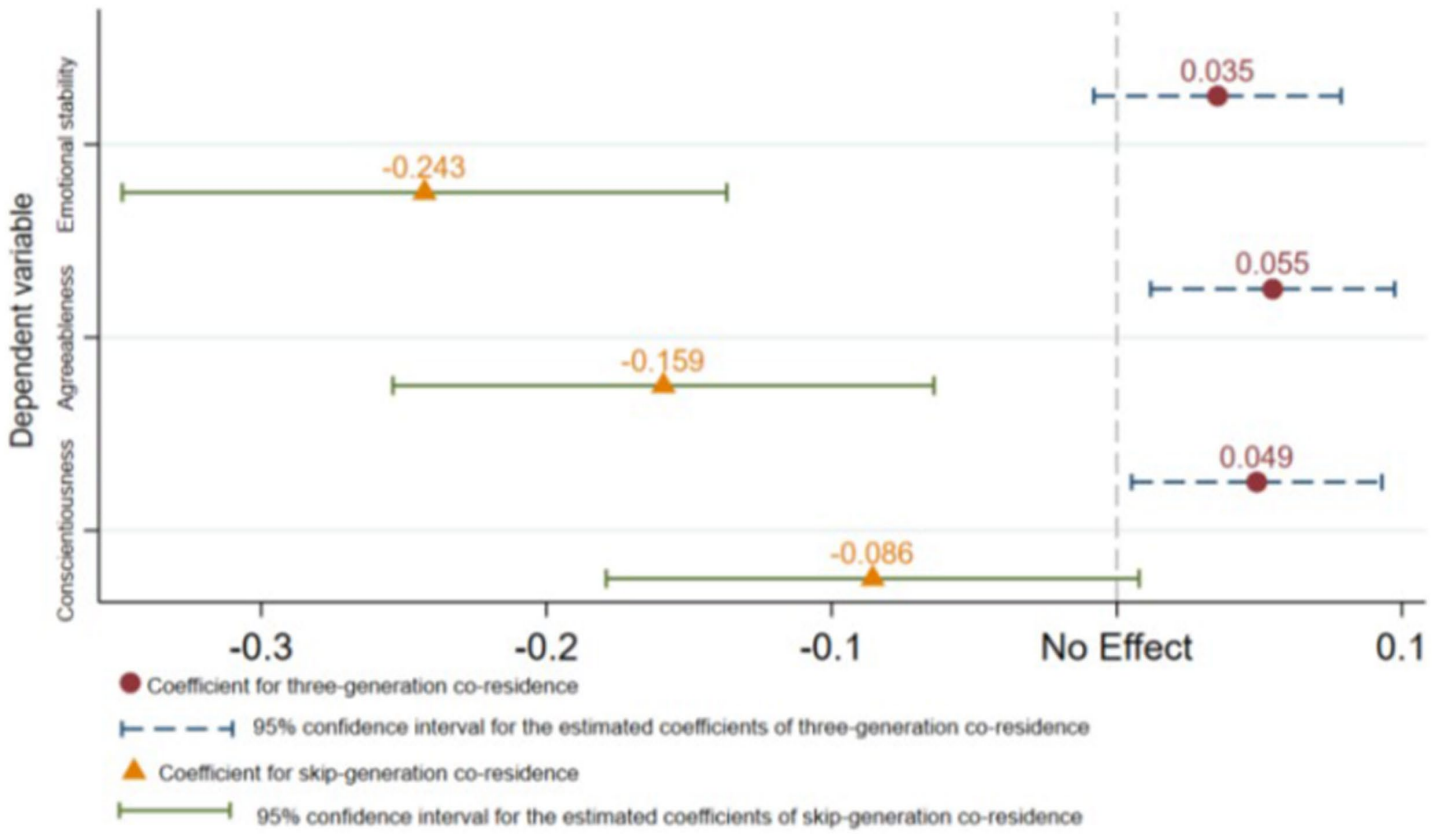
Estimation results of augmented inverse-probability weighting.

The results of the augmented inverse probability weighting (AIPW) method are presented in Figure 2. The vertical axis represents the dependent variable, while the horizontal axis depicts the magnitudes of impact coefficients. A vertical dashed line is used to indicate no impact. Triangular markers and a solid horizontal line represent the coefficient magnitudes and the 95% confidence interval, respectively, for assessing the impact of skip-generation co-residence on non-cognitive abilities in adolescents. Conversely, solid circles and dashed horizontal lines denote coefficient magnitudes and their corresponding 95% confidence intervals for evaluating the impact of three-generation co-residence on non-cognitive abilities in adolescents.

Figure 2 demonstrates that the emotional stability of adolescents is significantly positively impacted by three-generation co-residence (coefficient: 0.048), while skip-generation co-residence has a significant negative impact (coefficient: −0.221). Moreover, three-generation co-residence has a significant positive effect on the agreeableness of adolescents (coefficient: 0.063), whereas skip-generation co-residence has a significant negative effect (coefficient: −0.145). Additionally, three-generation co-residence has a significant positive influence on the conscientiousness of adolescents (coefficient: 0.041), while skip-generation co-residence does not have any notable impact (coefficient: −0.048); however, its direction is negative.

#### 4.3.2 Placebo testing

Although propensity score matching method eliminates the impact of observable variable differences between groups on the results and augmented inverse probability weighting method balances the impact of sample size differences between groups on the results, it still cannot control for the influence of unobservable variables on the results. In this paper, placebo testing is employed to mitigate the impact of unobservable variables on the results, a method widely used in relevant literature (Ferrara et al., 2012; Naoi et al., 2021).

Firstly, based on ordinary least squares estimation, the coefficient expressions of the impact of three-generation co-residence and skip-generation co-residence on adolescents’ non-cognitive abilities relative to two-parent co-residence are estimated as follows in Equation (6):


(6) $${\hat{\beta }}_{i}=\beta_{i}+\frac{COV\left(X,\;U|W\right)}{COV\left(X,\;X|W\right)}$$

The variable *W* includes all other control variables and X serves as an endogenous explanatory variable. The correlation between unobservable factors and endogenous explanatory variables is represented by $COV\left(X,\;U|W\right)$ . If $COV\left(X,\;U|W\right)$ is equal to zero, then the unobservable factors do not affect the estimation results, indicating that ${\hat{\beta }}_{i}$ is unbiasedness. However, this cannot be directly verified.

Therefore, in this paper, a theoretically irrelevant variable that would not affect the outcome variable is introduced as a substitute for the adolescents’ actual family residence structure. Specifically, a “fake family residence structure” is randomly assigned to individuals in the sample. For example, if an adolescent’s actual family residence structure is two-parent co-residence, they are assigned a random fake residence structure from the three family residence structures. Ordinary least squares regression is then conducted based on this setup, resulting in a biased ${{\hat{\beta }}_{i}}^{\mathrm{random}}$ . This estimating process is repeated 500 times to generate 500 ${{\hat{\beta }}_{i}}^{\mathrm{random}}$ and distribution of ${{\hat{\beta }}_{i}}^{\mathrm{random}}$ .

**Figure 3:**
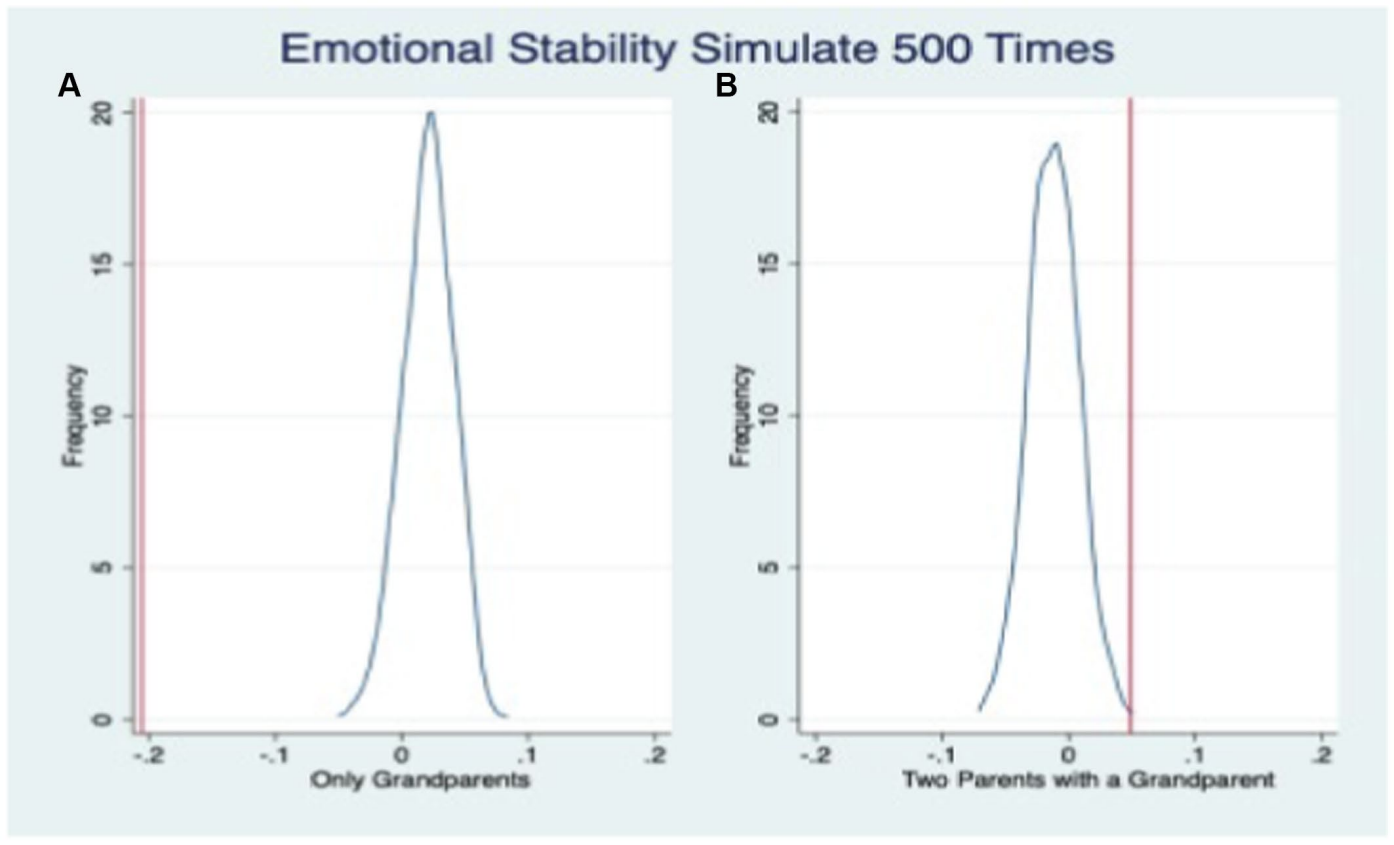
Placebo test: emotional stability.

**Figure 4:**
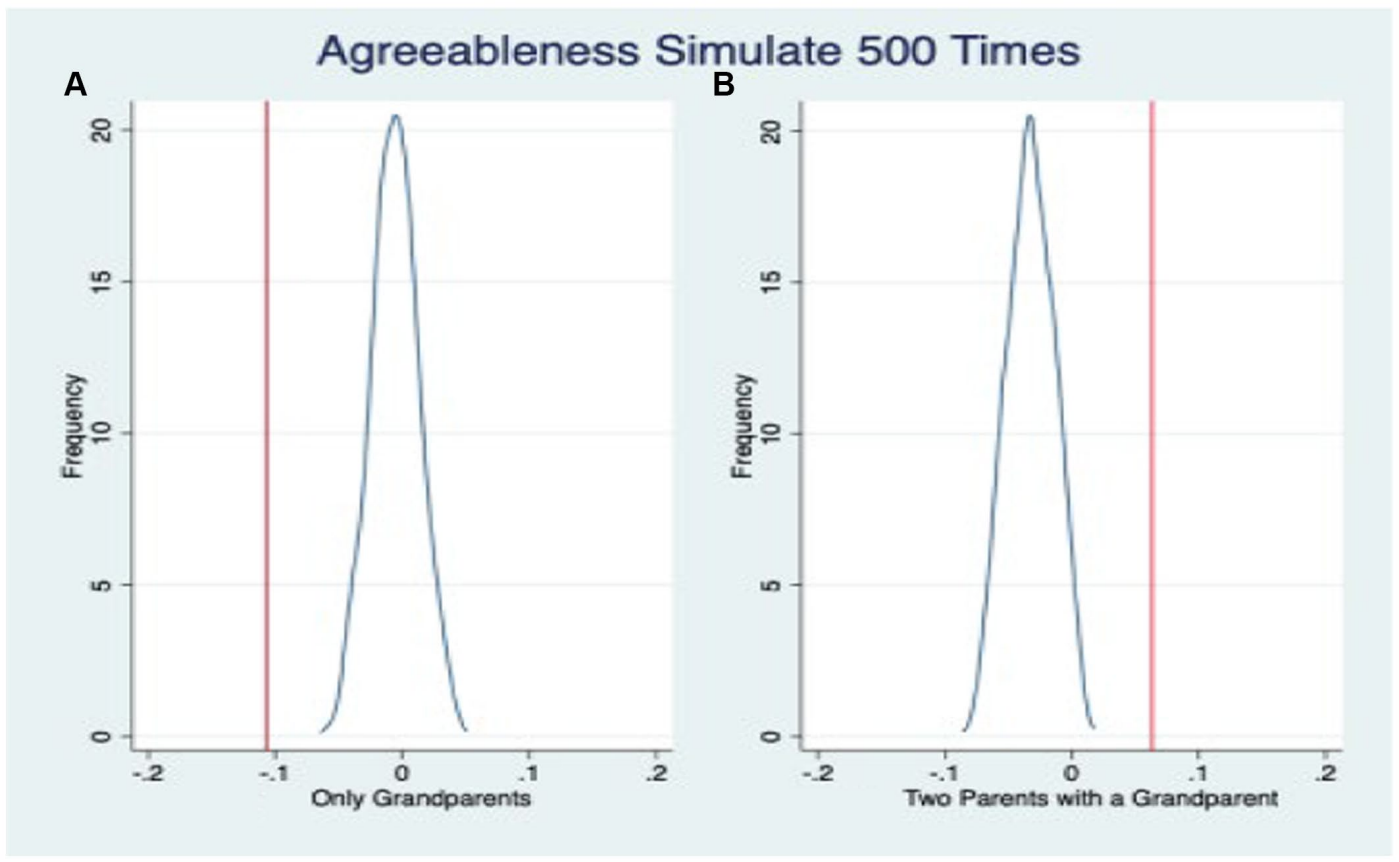
Placebo test: agreeableness.

**Figure 5:**
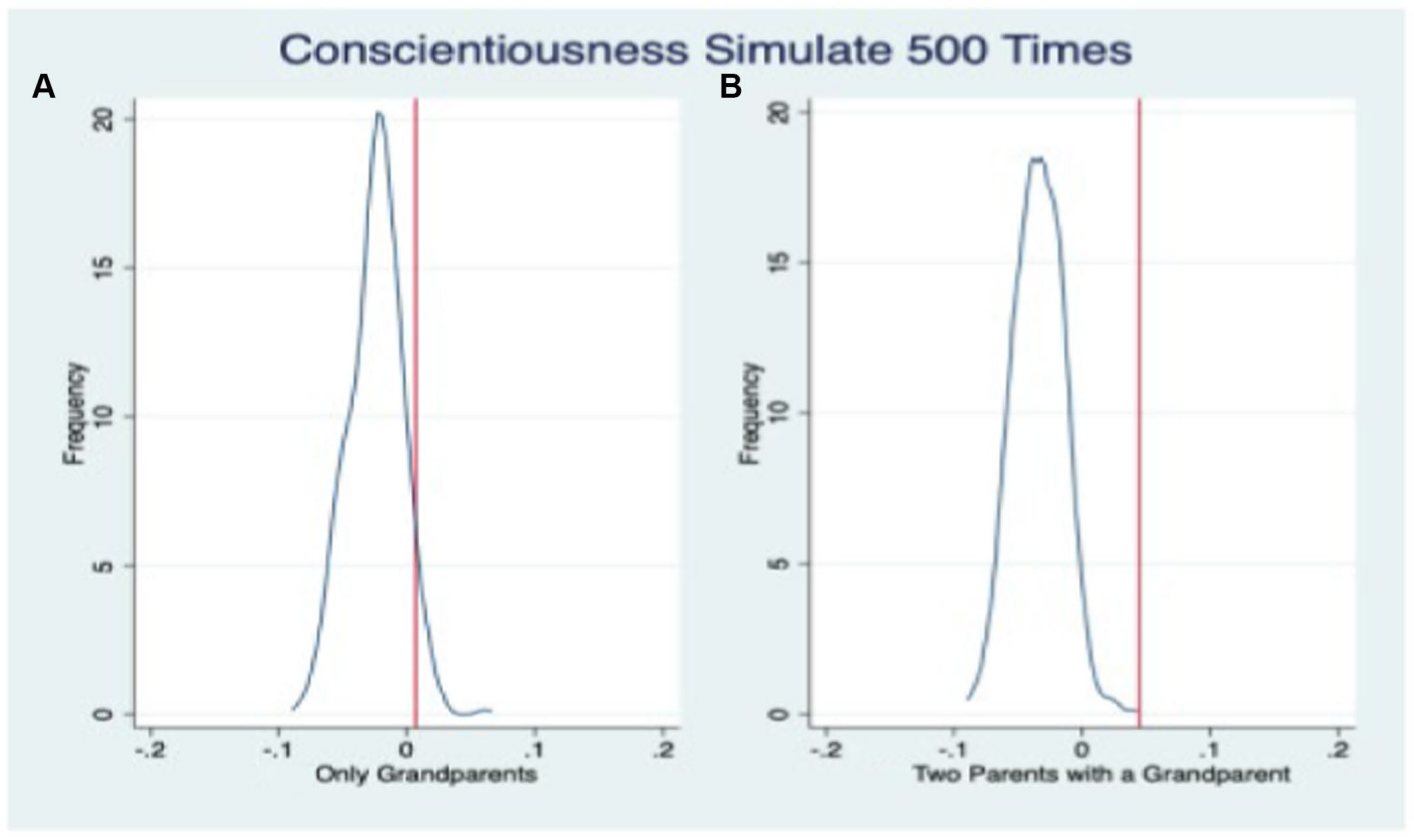
Placebo test: conscientiousness.

The placebo tests examining the impact of “fake family residence structure” on three dimensions of adolescents’ non-cognitive abilities are illustrated in Figures 3–5, respectively. The vertical line perpendicular to the x-axis represents the estimated coefficient from the ordinary least squares (OLS) regression analysis (i.e., the coefficient estimate presented in Table 6). The solid curve depicts the fitted distribution of coefficients in the placebo test. On the left side of the graph, we observe the coefficient distribution for skip-generation co-residence, while on the right side, we observe it for three-generation co-residence. Figures 3–5 demonstrate that the inclusion of the “fake family residence structure” does not exert a significant influence on the emotional stability, agreeableness, and conscientiousness of adolescents. The substantial coefficients obtained from the “actual” outcomes significantly differ from those derived from the placebo test’s “fake” results, thereby further affirming the robustness of our fundamental regression findings.

### 4.4 Heterogeneity analysis

Previous studies have demonstrated significant disparities in non-cognitive abilities among Chinese adolescents residing in urban and rural areas (Abbasi et al., 2022), as well as between genders (Gong et al., 2021). This section aims to investigate whether the influence of family residence structure on adolescents’ non-cognitive skills varies across different regions or genders.

#### 4.4.1 Gender dimension

The participants in this study consist of middle school students who are currently undergoing rapid physical and mental development. Notably, there exist significant disparities between boys and girls in terms of personality formation and various other aspects. Consequently, a gender-based heterogeneity analysis is conducted within this paper, with Table 11 presenting the influence of grandparent-grandchild co-residence on the non-cognitive abilities of adolescents belonging to different genders.

**Table 11:** Heterogeneity between male and female.

Variables	Emotional stability		Agreeableness		Conscientiousness	
	(1)	(2)	(3)	(4)	(5)	(6)
Gender	Male	Female	Male	Female	Male	Female
Three-generation co-residence	0.071**	0.031	0.053*	0.074***	0.008	0.074***
	(0.031)	(0.027)	(0.031)	(0.026)	(0.031)	(0.026)
Skip-generation co-residence	−0.160***	−0.208***	−0.103**	−0.113***	0.023	−0.009
	(0.048)	(0.044)	(0.048)	(0.043)	(0.047)	(0.044)
Constants	1.123***	1.228***	−0.155	0.114	1.851***	1.794***
	(0.174)	(0.155)	(0.174)	(0.150)	(0.172)	(0.154)
Observations	6,436	6,747	6,436	6,747	6,436	6,747
Adjusted *R* square	0.017	0.027	0.025	0.044	0.026	0.023
Control variables	Yes	Yes	Yes	Yes	Yes	Yes

*p < 0.1, **p < 0.05, ***p < 0.01. Standard errors are in brackets. The control variables in the regression include: age, minority, gender, household registration, preschool attendance, whether they are migrant children, whether they are only-children, boarding, and the highest level of education completed by their parents.

The first and second columns examine the heterogeneity of the impact of three-generation co-residence on adolescent emotional stability across genders. The findings suggest that three-generation co-residence positively influences boys’ emotional stability significantly, while it does not have a significant effect on girls’ emotional stability. Conversely, skip-generation co-residence negatively affects both boys and girls’ emotional stability significantly, with girls being more adversely affected than boys.

The fifth and sixth columns examine the heterogeneity of the impact of three-generation co-residence on adolescent conscientiousness across genders. The findings reveal a significant positive effect of three-generation co-residence on conscientiousness among girls, while no significant effect is observed among boys. Conversely, skip-generation co-residence from a different generation does not exert a significant influence on conscientiousness for either boys or girls.

#### 4.4.2 Household registration dimension

There is a binary economic system in China with a huge division between urban and rural areas, and there are significant differences in infrastructure and social services between urban and rural areas. However, it remains unclear whether there are notable discrepancies in the influence of family residence structures on the non-cognitive abilities of adolescents residing in urban and rural areas. Table 12 presents the impact of different family residence structures on the non-cognitive abilities of adolescents registered under agricultural and non-agricultural households, with columns 1, 3, and 5 representing the former group while columns 2, 4, and 6 represent the latter. The findings reveal that three-generation co-residence has a positive effect solely on emotional stability among adolescents from agricultural households; conversely, skip-generation co-residence negatively affects emotional stability for both agricultural and non-agricultural household registered adolescents. Additionally, three-generation co-residence positively influences agreeableness among both groups of adolescents; however, skip-generation co-residence significantly diminishes agreeableness primarily within agricultural household registered adolescents. Furthermore, three-generation co-residence predominantly impacts conscientiousness among samples from agricultural households whereas skip-generation co-residence does not exert any significant influence on conscientiousness for either agricultural or non-agricultural students.

**Table 12:** Heterogeneity between rural and urban.

Variables	Emotional Stability		Agreeableness		Conscientiousness	
	(1)	(2)	(3)	(4)	(5)	(6)
Household registration	Non-agricultural household	Agricultural household	Non-agricultural household	Agricultural household	Non-agricultural household	Agricultural household
Three-generation co-residence	0.042	0.051*	0.076***	0.046	0.023	0.069**
	(0.030)	(0.027)	(0.028)	(0.028)	(0.030)	(0.027)
Skip-generation co-residence	−0.169***	−0.186***	−0.076	−0.124***	−0.054	0.043
	(0.060)	(0.038)	(0.056)	(0.039)	(0.059)	(0.038)
Constants	1.561***	0.897***	0.156	−0.364**	1.772***	1.750***
	(0.175)	(0.155)	(0.163)	(0.162)	(0.172)	(0.155)
Observations	6,574	6,609	6,574	6,609	6,574	6,609
Adjusted R square	0.019	0.028	0.037	0.029	0.028	0.047
Control Variables	Yes	Yes	Yes	Yes	Yes	Yes

*p < 0.1, **p < 0.05, ***p < 0.01. Standard errors are in brackets. The control variables in the regression include: age, minority, gender, household registration, preschool attendance, whether they are migrant children, whether they are only-children, boarding, and the highest level of education completed by their parents.

### 4.5 Mechanism analysis

Why does three-generation co-residence contribute to the enhancement of non-cognitive abilities in adolescents? According to the Family Resource Theory, temporal and economic resources within families play crucial roles in shaping children’s human capital (Becker, 1991; Khanam and Nghiem, 2016). Regarding temporal resources, the involvement of grandparents in three-generation co-residence households indirectly influences adolescent development by fostering improved parent–child interactions. This is particularly evident when living with grandparents who assume caregiving responsibilities, enabling parents to allocate more leisure time toward their children’s growth and resulting in heightened parental engagement. Tan et al. (2021) discovered that grandparent involvement effectively reduces parental household chores, granting fathers within three-generation co-residence families additional time and energy for investing in their children’s development, thereby positively impacting adolescent progress. In terms of economic resources, research indicates that three-generation co-residence households possess significantly higher social capital compared to two-parent co-residence families due to the inclusion of an additional family unit (Zhang and Wu, 2020).

The previous analysis reveals significant disparities in parental involvement and family socioeconomic status across various family residence structures. Therefore, the objective of this study is to examine whether grandparent-grandchild residence arrangements impact students’ non-cognitive abilities by influencing parental involvement and family socioeconomic status. This paper measures four dimensions of parental involvement:

- *Parent–child communication*: The frequency at which parents engage in discussions with their children regarding “school events,” “relationships with classmates,” “relationships with teachers,” and “concerns or worries.” Responses are rated on a scale ranging from 1 (never) to 3 (often), and the mean score is calculated.
- *Parent–child companionship*: The frequency at which parents and children engage in shared activities such as eating meals together and watching television. Ratings range from 1 (never) to 6 (more than once a week).
- *Parent–child activities*: The frequency at which parents and children participate in joint activities such as reading together, visiting museums, zoos, science and technology museums, as well as attending performances, sports games, and movies. Ratings range from 1 (never) to 6 (more than once a week), with an average score computed.
- *Homework supervision*: The frequency at which parents check and guide their children’s homework on a weekly basis. Ratings range from 1 (never) to 4 (almost every day), with an average score calculated.

Family socioeconomic status is assessed based on respondents’ self-assessment of their current economic conditions within the family unit using a scale ranging from 1 (very difficult) to 5 (very affluent).

Table 13 presents the impact of various grandparent-grandchild residence structures on parental involvement across different dimensions, as depicted in columns 1 to 4. The findings suggest that three-generation co-residence outperforms two-parent co-residence families significantly in terms of parent–child communication, while it lags behind in parent–child companionship. No significant differences are observed between these family types regarding parent–child activities and homework supervision. However, skip-generation co-residence considerably diminishes parental involvement across all four dimensions compared to two-parent co-residence families. Column 5 illustrates the influence of diverse grandparent-grandchild living arrangements on family socioeconomic status. The results indicate that three-generation co-residence families exhibit a significantly higher socioeconomic status than two-parent families, whereas skip-generation co-residence is associated with a notably lower socioeconomic status compared to two-parent families.

**Table 13:** Impact of living with grandparents on parental involvement, family socioeconomic status.

	(1)	(2)	(3)	(4)	(5)
Variables	Parental involvement				Family socioeconomic status
	Parent–child communication	Parent–child companionship	Parent–child activities	Homework supervision	
Three-generation co-residence	0.034***	−0.037*	0.034	0.012	0.023**
	(0.010)	(0.019)	(0.027)	(0.020)	(0.011)
Skip-generation co-residence	−0.096***	−1.781***	−0.582***	−0.404***	−0.035*
	(0.017)	(0.030)	(0.042)	(0.033)	(0.018)
Age	−0.031***	−0.053***	−0.172***	−0.144***	−0.003
	(0.004)	(0.007)	(0.009)	(0.007)	(0.004)
Minority	−0.043***	−0.223***	−0.224***	−0.098***	−0.153***
	(0.017)	(0.030)	(0.042)	(0.032)	(0.018)
Gender	0.083***	0.023	0.031	−0.125***	0.017*
	(0.009)	(0.016)	(0.022)	(0.017)	(0.010)
Rural household registration	0.003	0.014	−0.097***	−0.012	−0.052***
	(0.010)	(0.019)	(0.026)	(0.020)	(0.011)
Preschool attendance	0.045***	0.058***	0.171***	−0.006	0.080***
	(0.012)	(0.021)	(0.029)	(0.023)	(0.013)
Migrant children	−0.006	0.056***	0.089***	−0.036	0.057***
	(0.012)	(0.021)	(0.030)	(0.023)	(0.013)
Only-child	0.085***	0.017	0.245***	0.125***	0.063***
	(0.010)	(0.018)	(0.026)	(0.020)	(0.011)
Boarding	0.075***	−0.063***	−0.195***	−0.174***	−0.108***
	(0.011)	(0.019)	(0.027)	(0.021)	(0.012)
Parents’ highest level of education	0.023***	0.007**	0.093***	0.039***	0.030***
	(0.002)	(0.003)	(0.004)	(0.003)	(0.002)
Constant	2.109***	6.174***	4.213***	4.021***	2.514***
	(0.060)	(0.108)	(0.152)	(0.118)	(0.066)
					
Observations	13,183	13,183	13,183	13,183	13,183
Adjusted *R* square	0.054	0.244	0.171	0.104	0.088

*p < 0.1, **p < 0.05, ***p < 0.01. Standard errors is in brackets. The control variables in the regression include: age, minority, gender, household registration, preschool attendance, whether they are migrant children, whether they are only-children, boarding, and the highest level of education completed by their parents.

## 5 Conclusions and discussions

Given the growing emphasis on the development of non-cognitive abilities in adolescents and the significant changes in family residence structures, it is crucial to investigate the impact of grandparent-grandchild co-residence arrangements on adolescents’ non-cognitive abilities. This study addresses this issue by examining grandparent-grandchild co-residence structures from a micro perspective. Using nationally representative data from the China Education Panel Survey conducted between 2013 and 2014, we employ ordinary least squares, propensity score matching, and augmented inverse probability weighting methods to analyze how grandparent-grandchild co-residence structures influence adolescents’ non-cognitive abilities. The following research findings are derived:

Firstly, the prevalence of intergenerational co-residence structures in Chinese families is noteworthy, with a significant number of adolescents residing with their grandparents. Among the entire sample, 56.68% of households consist of two parents living together, while a proportion of 26% involves adolescents living in grandparent-grandchild co-residence arrangements.

Secondly, based on previous research findings, this study classifies family residence structures into three distinct types: two-parent co-residence, three-generation co-residence, and skip-generation co-residence. It examines the influence of grandparent-grandchild co-residence arrangements on the non-cognitive abilities of adolescents. By controlling for individual characteristics such as gender, age, household registration status, minority background, preschool attendance history, migrant children status, only-child status, boarding arrangement, and parents’ highest educational attainment level, both ordinary least squares regression and propensity score matching methods consistently demonstrate that three-generation co-residence significantly enhances the non-cognitive abilities of adolescents. Conversely, skip-generation co-residence exhibits a detrimental impact on their non-cognitive abilities. These conclusions are further validated through augmented inverse probability weighting techniques and placebo tests. The findings of this study are further supported by existing literature. Li and Zhao (2017) demonstrate that in China, three generations co-residence has a significant positive impact on adolescents’ non-cognitive abilities, whereas skip-generation living arrangements have a negative effect. The research conclusions presented in this paper provide a crucial foundation for understanding the relationship between family structure and the non-cognitive development of adolescents. Similar cross-country studies have indicated that although variations exist among different countries (Anger, 2012; Elkins and Schurer, 2020), skip-generation co-residence may detrimentally affect adolescents’ non-cognitive skills (Radl et al., 2017).

Thirdly, the findings from the analysis of gender heterogeneity suggest that three-generation co-residence exerts a significant positive influence on boys’ emotional stability, while it has a notable positive impact on both boys’ and girls’ agreeableness. However, its effect on conscientiousness is only statistically significant among girls in our sample. In contrast, skip-generation co-residence demonstrates a substantial negative effect on both boys’ and girls’ emotional stability and agreeableness. Additionally, the results obtained from agricultural and non-agricultural heterogeneity analysis reveal that three-generation co-residence solely exhibits a significant positive impact on emotional stability for students with an agricultural household registration. Moreover, it significantly enhances the agreeableness of both agricultural and non-agricultural students; however, its influence on conscientiousness is only noteworthy among students with an agricultural household registration. Conversely, skip-generation co-residence negatively affects the emotional stability of both types of students and also diminishes their agreeableness specifically among those with an agricultural household registration.

Furthermore, this study posits that disparities in parental involvement and family economic circumstances can effectively account for the variations in the impact of three-generation co-residence, skip-generation co-residence, and two-parent co-residence on adolescents’ non-cognitive abilities. The study conducted by Anger and Schnitzlein (2017) also posited a similar explanatory mechanism, contending that the socioeconomic status of the family, parental educational attainment, and economic conditions all exert substantial influences on both cognitive and non-cognitive abilities in children. Specifically, three-generation co-residence significantly augments both parent–child communication frequency and the socioeconomic status of the family, thereby bolstering adolescents’ human capital through heightened parental engagement. Conversely, skip-generation co-residence’s adverse effects primarily stem from parental absence, leading to a notable decline in parent–child communication, bonding activities, supervision of homework tasks as well as a decrease in the family’s socioeconomic status.

Against the backdrop of accelerated industrialization and urbanization, it holds immense theoretical and practical significance to delve into the social consequences arising from family residence structures in order to comprehend current social development and address major real-life issues in China as well as developing countries. Despite the relaxation of birth restrictions allowing a third child, China is yet to establish a comprehensive social security system and domestic labor market. Grandparents are considered valuable contributors in mitigating caregiving deficits within families. While some studies have focused on the positive impact of grandparent involvement on China’s human capital accumulation from a macro model perspective, this paper provides corresponding evidence from a micro perspective, revealing that three-generation co-residence fosters human capital accumulation among adolescents while skip-generation co-residence significantly hampers various dimensions of adolescents’ non-cognitive abilities.

In the prevailing context of inadequate caregiving services, this paper empirically illustrates that grandparental involvement can significantly alleviate the shortage of care within families and positively influence adolescent development, especially when both parents are present. Nevertheless, in households where parents are absent, grandparental involvement, specifically in the form of skip-generation co-residence, cannot fully offset the detrimental effects of parental absence. Given the widespread practice of grandparent-grandchild co-residence, it becomes imperative to consider three-generation co-residence as a strategy to maximize family resources. The central challenge is to address the negative impacts associated with skip-generation co-residence. Based on these insights, the paper offers the following policy recommendations:

Firstly, strengthening family roles and intergenerational communication. In many developing countries, traditional extended family structures are gradually eroding due to economic pressures, migration, and urbanization, giving way to nuclear families or single-parent households. This transition can result in children growing up without adequate attention and care. Therefore, parents should be cognizant of their irreplaceable role in the upbringing of children and endeavor to provide essential emotional support and educational guidance amidst their busy schedules. Simultaneously, governmental bodies and social organizations should offer parental guidance programs and parent–child activities to assist parents in assuming their parenting responsibilities more effectively while fostering familial harmony and communication. Such external support is particularly crucial when parents are unable to spend extended periods with their children due to work commitments or other reasons.

Secondly, integrating social resources to facilitate intergenerational cohabitation. Given the prevailing resource disparities and inadequate social security systems in many developing nations, grandparents often assume a crucial caregiving role within families. However, acknowledging the challenges they may encounter while undertaking this responsibility is imperative. Therefore, it is essential to approach the issue of grandparents’ involvement in family care from a broader societal perspective and seek effective solutions. The government can promote and support three-generation living arrangements by formulating pertinent policies such as offering housing subsidies and tax incentives. This not only alleviates the burden on young parents but also fosters intergenerational communication and interaction. Furthermore, community organizations and social institutions play an indispensable role as well. For instance, organizing regular health check-ups for grandparents caring for their grandchildren or providing necessary medical assistance can be beneficial; alternatively, offering relevant training courses equips them with better coping mechanisms to address various challenges encountered during childcare.

In conclusion, enhancing family roles and intergenerational communication, as well as integrating social resources to support intergenerational cohabitation, can effectively address the challenges faced by developing countries in terms of family housing structure and the resultant non-cognitive development issues among adolescents, thereby promoting children’s healthy growth and fostering harmonious family development.

## Data availability statement

The original contributions presented in the study are included in the article/supplementary material, further inquiries can be directed to the corresponding authors.

## Ethics statement

Ethical review and approval was not required for the study on human participants in accordance with the local legislation and institutional requirements. Written informed consent from the patients/participants or patients/participants’ legal guardian/next of kin was not required to participate in this study in accordance with the national legislation and the institutional requirements.

## Author contributions

BT: Funding acquisition, Project administration, Supervision, Writing – original draft, Writing – review & editing. SX: Software, Validation, Visualization, Writing – original draft, Writing – review & editing. YZ: Conceptualization, Data curation, Formal analysis, Writing – original draft, Writing – review & editing. SL: Formal analysis, Software, Visualization, Writing – original draft, Writing – review & editing. XL: Resources, Validation, Writing – original draft, Writing – review & editing. HL: Funding acquisition, Project administration, Supervision, Writing – original draft, Writing – review & editing.
